# Reanalysis of the German PISA Data: A Comparison of Different Approaches for Trend Estimation With a Particular Emphasis on Mode Effects

**DOI:** 10.3389/fpsyg.2020.00884

**Published:** 2020-05-26

**Authors:** Alexander Robitzsch, Oliver Lüdtke, Frank Goldhammer, Ulf Kroehne, Olaf Köller

**Affiliations:** ^1^IPN – Leibniz Institute for Science and Mathematics Education, Kiel, Germany; ^2^Centre for International Student Assessment (ZIB), Kiel, Germany; ^3^DIPF – Leibniz Institute for Research and Information in Education, Frankfurt, Germany; ^4^Centre for International Student Assessment (ZIB), Frankfurt, Germany

**Keywords:** educational measurement, large-scale assessment, mode effects, scaling, linking

## Abstract

International large-scale assessments, such as the Program for International Student Assessment (PISA), are conducted to provide information on the effectiveness of education systems. In PISA, the target population of 15-year-old students is assessed every 3 years. Trends show whether competencies have changed in the countries between PISA cycles. In order to provide valid trend estimates, it is desirable to retain the same test conditions and statistical methods in all PISA cycles. In PISA 2015, however, the test mode changed from paper-based to computer-based tests, and the scaling method was changed. In this paper, we investigate the effects of these changes on trend estimation in PISA using German data from all PISA cycles (2000–2015). Our findings suggest that the change from paper-based to computer-based tests could have a severe impact on trend estimation but that the change of the scaling model did not substantially change the trend estimates.

## Introduction

Since 2000, the Program for International Student Assessment (PISA; OECD, 2016; Reiss et al., 2016) has assessed the competencies of 15-year-old students in the domains of mathematics, reading, and science in a 3-year cycle. Based on a literacy concept (Literacy; OECD, 2016), it is assumed that sufficient competencies in the three areas tested are necessary prerequisites for vocational and social participation (see OECD, 2016) and that national education systems should offer learning opportunities in which children and young people can develop the corresponding competencies. In this sense, PISA should also be an instrument for assessing the performance of education systems. The performance of a participating country can be determined by social comparison, for example, with the mean value of all Organization for Economic Co-operation and Development (OECD) countries. The PISA cycle with recurring tests (every 3 years), however, allows the assessment of trends in the performance of 15-year-olds for each participating country. For example, the disappointing performance of German students in PISA 2000 (Baumert et al., 2001) resulted in far-reaching measures to improve the quality of education in Germany (Waldow, 2009; Klieme et al., 2010). In subsequent PISA studies, the performance of German 15-year-olds in reading, mathematics, and science rose continuously. Whereas in PISA 2000, performance in all three domains was significantly below the OECD average, in PISA 2012, German students performed significantly above the OECD average (see Prenzel et al., 2013). This gain was interpreted, at least in terms of education policy, as a consequence of successful reforms in the education system, for example, structural reforms such as the introduction of all-day schools, reading interventions for low-achieving students in elementary and secondary schools, and intervention programs in early childhood education (Ringarp, 2016; Niemann et al., 2017).

In PISA 2015, however, this positive trend for Germany did not continue for mathematics and science. Average performance in science, which showed the most pronounced decrease, dropped by 15 points within 3 years (2012: 524 points; 2015: 509 points). This dramatic drop needs further explanation if one follows the argument that changes in average performance at the country level over relatively short periods are typically rather small when the test conditions are kept constant (Beaton and Zwick, 1990; Mazzeo and von Davier, 2008, 2014). Interestingly, the international trend (across all participating OECD countries) also showed a drop of eight points in average science performance from 2012 to 2015. This raises the question of whether a performance decline across 3 years reflects an actual decline in scientific competence or whether this decline can (at least in part) be attributed to the many changes implemented in the PISA 2015 study. In comparison with the five previous PISA cycles (PISA 2000, 2003, 2006, 2009, 2012), several substantial changes were implemented in the administration and analysis of PISA 2015 (for an overview of changes, see OECD, 2016, Annex 5). In this article, we focus on two substantial changes (but see Jerrim et al., 2018b, for a broad discussion of other changes). First, instead of a one-parameter logistic (1PL) model (Rasch, 1960), in which only the difficulty parameters for the items are estimated, a two-parameter logistic (2PL) model (Birnbaum, 1968), which estimates an additional discrimination parameter for each item, was used to scale the data. Second, PISA 2015 switched from paper-based assessment (PBA) to computer-based assessment (CBA) in all three competence domains.

In this article, we investigate whether the average performance differences between PISA 2012 and PISA 2015 might not reflect a decrease in the performance of the German school system but, instead, could have been caused by the switch of the test mode – from PBA to CBA – or by the change of the scaling model (1PL vs. 2PL). To this end, we re-analyzed the national PISA data of all six PISA studies from 2000 to 2015. We also took into account the German data from the 2014 field test for PISA 2015 in which test administration effects (PBA vs. CBA) were experimentally assessed in a randomized between-subjects design.

### The Program for International Student Assessment

PISA is an OECD study designed to provide OECD members and partner countries with indicator-based information on the performance of their education systems every 3 years. The target population in each country is the 15-year-old students. School attendance is still compulsory for this age group so that the tests fully reflect the age group in its heterogeneity. The primary indicators in PISA are students’ performance in mathematics, reading in the language of instruction, and science. In all three competency domains, students are primarily taught at school, and curricular goals show substantial overlap across countries. The test frameworks of the three test domains are based on the Anglo-Saxon functional literacy concept (OECD, 2019). In the context of PISA, the term *functional* mainly comprises two aspects, namely, applicability for current and later (i.e., post-school) participation in a culture, and connectivity in the sense of continuous learning throughout one’s lifetime.

In the PISA test design, the number of items administered in each of the three competence domains differs considerably across PISA cycles; the major domain comprises about half of the administered items, and the two minor domains share the second half. In PISA 2000 and PISA 2009, reading was the major domain. In PISA 2003 and 2012, it was mathematics, and in PISA 2006, it was science. Starting with PISA 2015, the aim was to increasingly balance the extent to which the number of administered items differed across the three domains, while the division into one major and two minor domains was retained. In PISA 2015, science was the major domain for the second time after PISA 2006.

In general, PISA uses *link items*, which are administered in several studies. Using a set of common link items across different time points ensures that a common metric can be established over time. Hence, the performance of 15-year-old students in countries can be compared across the different PISA studies (von Davier A. A. et al., 2006; Kolen and Brennan, 2014), and trend estimates can be used to check whether the performance of education systems has improved or declined. In the following, the methodological challenges related to the estimation and interpretation of these trends are discussed.

### Computation of Trend Estimates in PISA

In the literature on trend analysis in international large-scale assessments, the original trend is distinguished from a marginal trend (Gebhardt and Adams, 2007; see also Carstensen, 2013; Sachse et al., 2016). In the original trend estimate, the change in the average performance of a participating country is computed using item response models that employ international item parameters. For this purpose, the international item parameters that are obtained in each PISA study and are based on all participating countries are linked (Gebhardt and Adams, 2007) or concurrently scaled (OECD, 2017) to the common PISA metric. For original trend estimation, a reference study has to be chosen: the PISA 2000 study is used for reading, PISA 2003 is used for mathematics, and PISA 2006 is used for science (these are the cycles in which the respective domain was a major domain for the first time). In these studies, the ability distribution comprising all participating students in all countries in a corresponding PISA study is fixed at a mean of 500 and a standard deviation of 100.

On the other hand, the estimation of the marginal trend (i.e., national trend) for a participating country is based only on the data and item parameters of the respective country. First, the national item parameters are estimated from the separate PISA studies of the participating country. Then, these item parameters are linked to a common metric (Gebhardt and Adams, 2007). Hence, the national trend estimate is determined only by the link items that are administered across different PISA studies. In contrast to the original trend estimate, items that are used only for one study (non-link items) do not influence the marginal trend estimate. As a reference for the marginal trend estimate in a domain, the mean and the standard deviation of the first study of the participating country are usually fixed (e.g., for reading in Germany in PISA 2000: *M* = 484, SD = 111).

In official publications on the PISA study (e.g., OECD, 2014), the original trend estimates are usually computed by subtracting the cross-sectional country mean of the first time point from the second time point. However, reanalyses have shown that the original trend estimates can deviate considerably from the marginal trend estimates (Gebhardt and Adams, 2007; Carstensen, 2013; Robitzsch and Lüdtke, 2019). These differences between original and marginal trends can be attributed to cross-sectional differential item functioning (country DIF) and the test design of the PISA study (Monseur et al., 2008). Country DIF describes the fact that national and international item parameters differ. Thus, the difference between the mean of a participating country and the international mean (across all countries) depends on which items are selected for the comparison. This poses a particular challenge for trend estimation because major and minor domains change between PISA studies (Mazzeo and von Davier, 2014). In the case that a competency domain is a major domain in a PISA cycle, a relatively large number of non-link items is administered for this domain in addition to a comparably smaller number of link items. It can be the case that the average country DIF for the link items for a participating country differs from the average country DIF for the non-link items. Then, the country mean, based on the link items, may differ from the mean obtained from the international metric, which is computed from all items (i.e., link items and non-link items). As a consequence, the original trend estimate deviates from the marginal trend estimate, because the latter only takes link items into account (Monseur et al., 2008; Sachse and Haag, 2017).

When estimating trends, two primary sources of uncertainty need to be considered (Wu, 2010). First, each estimate of a country mean contains an estimation error concerning the population of students because, in each PISA study, only a sample of students is taken from each participating country. Second, the mean of a participating country could be larger or smaller depending on the selection of items for a PISA cycle. Thus, it could be argued that the choice of items – in addition to the selection of students – represents an additional source of uncertainty that should be taken into account in the statistical quantification of the error of trend estimates (Cronbach et al., 1963; Husek and Sirotnik, 1967; Brennan, 1992; Michaelides and Haertel, 2004, 2014; Monseur and Berezner, 2007; Michaelides, 2010; Lu et al., 2015). For the original trend, PISA quantifies the variability in the trend estimate caused by items as a *link error* (Monseur and Berezner, 2007; OECD, 2014). This link error quantifies the variability of the international parameters of the link items across several PISA studies and completely ignores the selection of non-link items. Up until PISA 2015, country DIF was explicitly not included in the calculation of this link error. However, Monseur and Berezner (2007) demonstrated in a reanalysis of PISA data that the uncertainty in the original trend estimate is underestimated by the officially reported link error because country DIF is ignored. Since PISA 2015, a different method for the computation of link error is in operational use, which has the potential to include country DIF as an additional source of uncertainty (OECD, 2017).

It has been argued that, to obtain reliable estimates of original trends, a moderately large number of link items is required in order to reduce the variability caused by country DIF (Mazzeo and von Davier, 2008; Monseur et al., 2008). If this is not the case (i.e., a relatively small number of link times is used) as, for example, in the domain of reading in the PISA studies from 2000 to 2009, marginal trend estimates can lead to more robust, efficient, and less distorted assessments of competencies over time than original trend estimates (Gebhardt and Adams, 2007; Carstensen, 2013; Sachse et al., 2016; Robitzsch and Lüdtke, 2019).

Since PISA 2015, the statistical approach used in PISA has been based on the assumption of partial invariance of items across all countries and PISA cycles (von Davier et al., 2019b). The item parameters for individual countries are assumed to be non-invariant across countries only if the country DIF is quite large (Oliveri and von Davier, 2011; OECD, 2017; von Davier et al., 2019b). In this case, the item parameters for a country are freely estimated and could, therefore, deviate from the common international item parameters. The comparison of a participating country with an international reference is then based only on those items that are assumed to be invariant, and items with too large deviations (country DIF) are removed from the linking. It is important to emphasize that the source of variability of the selection of link items even remains when all item parameters are assumed to be invariant because the data-generating model likely contains items possessing non-invariant parameters. It has been argued that the scaling under partial invariance in comparison to linking under full non-invariance (i.e., national parameters are used for all countries and items) results in more efficient trend estimates (Oliveri and von Davier, 2014; OECD, 2017; von Davier et al., 2019b). Sachse et al. (2016) showed in a simulation study that marginal trend estimates were more efficient than original trend estimates if country DIF existed. Moreover. if country DIF effects were normally distributed, excluding items with country DIF from the linking of a country to the common metric turned out to be less efficient than using an original trend estimate based on all items (see also Robitzsch and Lüdtke, 2019, for further simulation evidence).

In summary, it is evident that the estimation and interpretation of trends in PISA is challenging (Mazzeo and von Davier, 2008, 2014), even if the test administration conditions remain constant across the different studies. Therefore, it can be concluded that substantial changes in the administration conditions are likely to lead to less stable trend estimates, which makes it even more challenging to interpret trends in competencies across time. In this paper, marginal trend estimation is used to estimate the impact of two primary changes made in PISA 2015 on the trend for 15-year-olds in Germany.

### Changes in PISA 2015

In the following, two critical changes implemented in the PISA 2015 study compared to the previous five studies (2000, 2003, 2006, 2009, and 2012) are discussed: the change of the scaling model and the switch from PBA to CBA.

#### Change of the Scaling Model

Large-scale assessment studies differ in their choice of the scaling model used for cognitive item responses. For example, the PISA study used a 1PL model to scale the competency test up until 2012. Other studies, on the other hand, used the 3PL model, which provides an item difficulty parameter, a discrimination parameter, and a guessing parameter for each item, for example, the Trends in International Mathematics and Science Study (TIMSS; Martin et al., 2016), the Progress in International Reading Literacy Study (PIRLS; Martin et al., 2017), or the National Assessment of Educational Progress (NAEP; National Center for Education Statistics [NCES], 2013). A 2PL model, which postulates a difficulty and a discrimination parameter for each item, was used in PIAAC (Program for the International Assessment of Adult Competencies; Yamamoto et al., 2013) and has also been used since 2015 in PISA to scale the performance tests (OECD, 2017).

To justify the choice of the 2PL or 3PL model instead of the 1PL model, the psychometric literature often refers to a better model fit (i.e., a better fit of the item response function; Oliveri and von Davier, 2011, 2014; von Davier et al., 2019b). Especially if items with different response formats (e.g., multiple-choice and constructed-response formats) are administered, items are modeled to possess different reliabilities, which, in turn, leads to different discrimination parameters and a better model fit of the 2PL model (Mazzeo and von Davier, 2008). The 3PL model has the additional advantage that guessing behavior can be modeled for multiple-choice items, which often leads to a better model fit in large-scale assessments compared to the 2PL model (Aitkin and Aitkin, 2011).

Empirically, however, the question arises as to how strongly findings differ if a 1PL or 2PL model is used to scale the performance data. Macaskill (2008) used PISA data from PISA 2003 and PISA 2006 and compared country means and country trend estimates under both the 1PL and 2PL models. For PISA 2006, the absolute differences between the country means obtained by the 1PL and 2PL models were relatively small on average and the correlations between the country means from the 1PL and 2PL models were high, even though for a few countries, larger deviations (especially in reading) were observed. Furthermore, for PISA 2015 data, Jerrim et al. (2018b) found negligible differences between the relative order of country means for the 1PL model and the 2PL model. For the TIMSS 1995 data set, the 1PL and the 3PL models were compared and the rank order in the country means was found to be very consistent (Brown et al., 2007). However, the country means substantially differed for low-performing countries. Based on these results, it could be assumed that a change of the scaling model from 1PL (PISA 2012) to 2PL (PISA 2015) would not lead to significantly different trend estimates.

#### Change in the Mode of Test Administration

From a diagnostic point of view, reasons for switching the mode from paper (PBA) to computer (CBA) can be to implement innovative task formats (Parshall et al., 2010), to increase measurement efficiency (van der Linden, 2005), or to collect process data in addition to response data (Goldhammer et al., 2017; Kroehne and Goldhammer, 2018). From a psychometric point of view, a change of the mode of test administration between different assessments poses the challenge of ensuring the comparability of measurements between different modes, because otherwise, a valid trend estimation cannot be obtained (Mazzeo and von Davier, 2008).

The crucial question of whether the change of mode influences the psychometric properties of the measurement (mode effect; see Kroehne and Martens, 2011) has already been investigated in the context of international large-scale assessments in PIAAC 2012 (Yamamoto, 2012; OECD, 2013). It also needs to be added that studies of the International Association for the Evaluation of Educational Achievement (IEA) are currently implementing CBA components (ePIRLS 2016; Martin et al., 2017) or switching from PBA to CBA (eTIMSS 2019; Fishbein et al., 2018).

Meta-analyses have shown that the direction and strength of the mode effect could depend on different factors, for example, the subject area, the type of test composition (see Wang et al., 2008; Kingston, 2009), or the dependence on the response format (Bennett et al., 2008). As a consequence, it has been argued that a separate examination of mode effects is required for each study, insofar as mode effects are assumed to be the result of an unknown mixture of diverse effects of changes in measurement properties (Kroehne and Martens, 2011).

In the PISA 2015 field test study, mode effects were tested by randomly assigning students of a school to either CBA or PBA tasks (OECD, 2017). In this case, the two groups referring to the PBA and CBA test condition can be assumed to be randomly equivalent. Hence, differences in test performance can be attributed to differences in mode (CBA vs. PBA). The analysis of the field test data of all participating countries showed that in a 2PL model, the item discrimination between modes varied only slightly, but there were mode effects with regard to item difficulties (OECD, 2017; see also Kroehne et al., 2019a). Overall, the CBA items proved to be more difficult. On the basis of the assumption that only a subset of items contributed to this average mode effect at the test level, a common scale was established that consisted of CBA items that did not show any change in difficulty compared to PBA (invariant items). CBA items with a mode effect on item difficulty (non-invariant items), on the other hand, were allowed to differ from PBA items in item difficulties. To obtain credible trend estimates, it is crucial that all items with a mode effect (i.e., the item difficulties of a CBA and PBA version of an item differ) are declared to be non-invariant. Also, there is an unverified assumption in the international analyses of the field test that the mode of test administration does not have an interaction with the participating country (OECD, 2017; see also Jerrim et al., 2018a).

## Research Questions

This article investigates whether internationally reported original trend estimates for Germany in the three competency domains (science, mathematics, and reading) can be replicated with the marginal trend estimates based on the German samples from the PISA studies conducted since PISA 2000. We focus on the following research questions.

### Mode Effects

In a first step, we investigated how trend estimates changed due to the switch in test administration mode in 2015. From the research literature (e.g., Kroehne and Martens, 2011), it is known that the direction of mode effects, that is, whether a test mode makes tasks easier or more difficult, is not clear. Thus, any switch from PBA to CBA should be accompanied by an empirical study that makes it possible to estimate the magnitude of the mode effect. Such a mode-effect study was carried out in the participating countries of the PISA 2015 field test. The analysis of the field test, comprising all participating countries, resulted in a subset of invariant items in all three domains (science, mathematics, and reading), which made it possible to control for the mode effect in the PISA 2015 main study. However, this approach ignored country-by-mode interaction effects, that is, it made the crucial (but unverified) assumption that the mode effects were identical for all participating countries. We further pursued this issue and investigated how the OECD approach to possible mode effects could have influenced trend estimates for Germany.

### Change of the Scaling Model

Second, the extent to which trend estimates in PISA depend on the choice of the scaling model (1PL vs. 2PL) was analyzed. While the scaling in the PISA studies 2000–2012 was conducted using the 1PL model, the 2PL model was used for the first time in PISA 2015. The latter usually produces a better model fit. Still, it non-uniformly weights the items in the ability estimate, whereas the items in the 1PL model enter ability estimation with uniform weighting. Using these different scaling approaches, we examined whether these different scaling models result in differing trend estimates.

### Differences Between Marginal and Original Trend Estimates

Finally, we investigated the extent to which the trend estimates differ if the analyses do not refer to the international data sets (original trend) but are instead restricted to the German data sets (marginal trend). In the literature, it has been shown that considerable deviations can occur if the items used for trend estimation show DIF in individual countries (e.g., Monseur et al., 2008).

## Study 1: Investigating Mode Effects Using German Field Test Data for PISA 2015

In the PISA 2015 field test, students in a school were randomly assigned to a paper task (PBA) or a computer task (CBA) condition using the same set of items. We used the German sample of the field test to test whether mode effects in Germany could be observed for the domains of science, mathematics, and reading.

### Methods

The analysis was based on a subsample of the PISA 2015 field test conducted in Germany in spring 2014 with *N* = 517 students in PBA mode and *N* = 506 students in CBA mode. The students within the 39 schools were randomly assigned to the PBA or CBA condition. Each participant worked on items in two of the three domains (e.g., science and reading). Due to the random assignment to the conditions, any differences in test performance can be attributed to differences in mode (CBA vs. PBA).

The item responses in the field test study were scaled using a 1PL model for dichotomous and polytomous items (partial credit model; Masters and Wright, 1997). The sample size proved to be too small for the estimation of a 2PL model. The sample sizes per item ranged between *N* = 108 and *N* = 125 (mean *N* = 116.9) in the PBA mode and between *N* = 96 and *N* = 115 (mean *N* = 108.4) in the CBA mode. The samples of the two administration modes (CBA and PBA) were first scaled separately. The average mode effect was calculated by using a subsequent mean-mean linking of the item difficulties (Kolen and Brennan, 2014). The effect size of the mode effect *d* for a competency domain was determined by dividing the mean difference of the CBA and PBA mode by the standard deviation of the corresponding competence distribution in the PBA mode. Furthermore, the standard deviation for the difference in item difficulties between the two test modes was computed (DIF standard deviation, SD_*mode*_; Camilli and Penfield, 1997). The standard errors were calculated using a double jackknife method (Xu and von Davier, 2010; see also Haberman et al., 2009), which takes into account both the uncertainty associated with the sampling of students and the uncertainty associated with items. The 39 schools were used as jackknife zones for computing the standard error associated with the sampling persons. Testlets were used as jackknife zones for the assessment of uncertainty associated with the sampling of items, as individual items were often administered with a common stimulus (testlets; Monseur and Berezner, 2007). In total, 28 testlets in science, 38 testlets in mathematics, and 24 testlets in reading were used as jackknife zones. The jackknife method also provides a bias correction of estimators (Cameron and Trivedi, 2005; Hsieh et al., 2010).

The OECD did not carry out a country-specific analysis of the PISA 2015 field test data; they only conducted analyses in which the data of all countries were combined (OECD, 2017). In these analyses, items were identified that had the same statistical properties under the CBA and PBA conditions (i.e., invariant items). These invariant items were assumed to not be affected – at least at the international level – by a mode effect. Motivated by this approach, we carried out a mean–mean linking of the item difficulties (Kolen and Brennan, 2014) for the German field test data under two conditions. In a first analysis, all items of a competency domain were considered for the mean–mean linking approach, that is, also those items that were identified as non-invariant in the evaluation of the international sample of the field test data (“all items”). In a second analysis, linking for each domain was carried out only on the items that were declared to be invariant by the OECD (“invariant items”). Based on the findings, it was possible to check the extent to which items that were identified as invariant in the international analysis were affected by a mode effect that was specific to the German field test sample. The software R (R Core Team, 2019), as well as the R packages TAM (Robitzsch et al., 2019) and sirt (Robitzsch, 2019), was used for all statistical analyses.

### Results

Table 1 shows the results for the test of a mode effect in the three domains of science, mathematics, and reading for the German field test of PISA 2015. First, the results are presented based on all items (“all items”) administered in the field test. For all three competency domains, the CBA mode had a negative effect compared to the PBA mode, that is, tasks on the computer were more difficult than on paper. The mode effect in science and mathematics was significantly different from zero. Overall, the effect sizes of the mode effects were substantial: *d* = −0.23 (science), *d* = −0.14 (mathematics), and *d* = −0.13 (reading). Differences in the mode effects between the competency domains were not statistically significant (Wald test: χ^2^ = 1.39, *df* = 2, *p* = 0.50); that is, the mode effect was independent of the competence domain investigated. Furthermore, the standard deviation of the item-specific mode effects (SD_*mode*_) revealed that the difference in item difficulties between the two test modes varied considerably across items. Thus, the change of the test mode did not induce a constant shift in the item difficulties (see also OECD, 2017) but rather affected items differently. It became clear that the variability of item difficulties attributable to differential mode effects was particularly pronounced for the domain of reading and was relatively weak for science (see Camilli and Penfield, 1997, for an effect size classification).

**TABLE 1:** Results of the German field test data 2014 for the mode effect, based on the 1PL Model.

	All items							Invariant items				
Domain	*N*			*d*		SD_*mode*_			*d*		SD_*mode*_	
	PBA	CBA	*I*	*Est*	SE	*Est*	SE	*I*	*Est*	SE	*Est*	SE
Science	340	338	77	**−0.23**	0.08	0.17	0.05	56	**−0.17**	0.08	0.13	0.06
Mathematics	345	340	66	**−0.14**	0.07	0.31	0.05	36	−0.09	0.07	0.27	0.08
Reading	349	334	82	−0.13	0.10	0.43	0.05	47	−0.06	0.09	0.25	0.07

*N = number of students; I = number of items; PBA = paper-based assessment; CBA = computer-based assessment; d = effect size for mode effect CBA vs. PBA (negative effect: disadvantages of CBA); Est = estimate; SE = standard error; SD_mode_ = standard deviation of the difference between item difficulties in CBA and PBA mode; Statistically significant (p < 0.05) d values are printed in bold.*

In a second analysis, only the items declared to be invariant by the OECD were considered in the linking. The effects of the test mode were still apparent, even though they were somewhat weaker and only significantly different from zero for science (science: *d* = −0.17, mathematics: *d* = −0.09, reading: *d* = −0.06). These findings suggest that mode effects were not fully adjusted based on the invariant items selected by the OECD, at least for Germany. The standard deviations of the item-specific mode effects were somewhat smaller but remained significant in the domains of mathematics and reading.

## Study 2: Trend Estimates in PISA for Germany, Based on Scaling Approaches at a National Level

In the following, we examine the sensitivity of the trend estimates for the German PISA sample with respect to the change of the scaling model and the change of the test administration mode in PISA 2015. For all three competency domains, the German samples of the PISA studies were scaled using different approaches, taking the mode effect that was identified in the field test study for PISA 2015 into account and also not taking it into account.

### Methods

Table 2 provides an overview of the German PISA samples that were used in the analyses. The fourth column reports the number of students to whom items in a domain were administered in a particular PISA study. It should be noted that the items in a domain were only administered to all students in a study if it was the major domain. In addition, the total number of items administered in a domain for a study is listed in the fifth column. A subset of these items was used as link items in our analysis. For example, 103 items were presented in science in PISA 2006, of which 77 items were used as link items. For an item to be a link item, it must have been administered in at least two PISA studies.

**TABLE 2:** Sample sizes of PISA studies used for linking study.

Domain	Study	Mode	*N*	#Items	#Link items	
					All	Invariant
Science	2006	PBA	4881	103	77	56
	2009	PBA	3477	53	52	40
	2012	PBA	3505	52	52	40
	2014	PBA	340	91	77	56
	2014	CBA	338	91	77	56
	2015	CBA	6501	181	77	56
Mathematics	2003	PBA	4656	84	31	16
	2006	PBA	3795	48	31	16
	2009	PBA	3503	35	31	16
	2012	PBA	4971	84	66	36
	2014	PBA	345	70	66	36
	2014	CBA	340	68	66	36
	2015	CBA	2739	69	66	36
Reading	2000	PBA	5060	128	35	19
	2003	PBA	2555	27	24	13
	2006	PBA	2701	28	24	13
	2009	PBA	4975	100	82	47
	2012	PBA	3470	43	42	23
	2014	PBA	349	85	82	47
	2014	CBA	334	85	82	47
	2015	CBA	2746	87	82	47

*Study = PISA study used. “2014” denotes the field test for PISA 2015, which was conducted in spring 2014. Mode = administration mode; PBA = paper-based assessment; CBA = computer-based assessment; N = sample size; #Items = number of items used in scaling model; #Link items = number of link items used in joint linking. The column “invariant” denotes the number of items that were declared to be invariant across CBA and PBA modes, according to the OECD (2017).*

**FIGURE 1:**
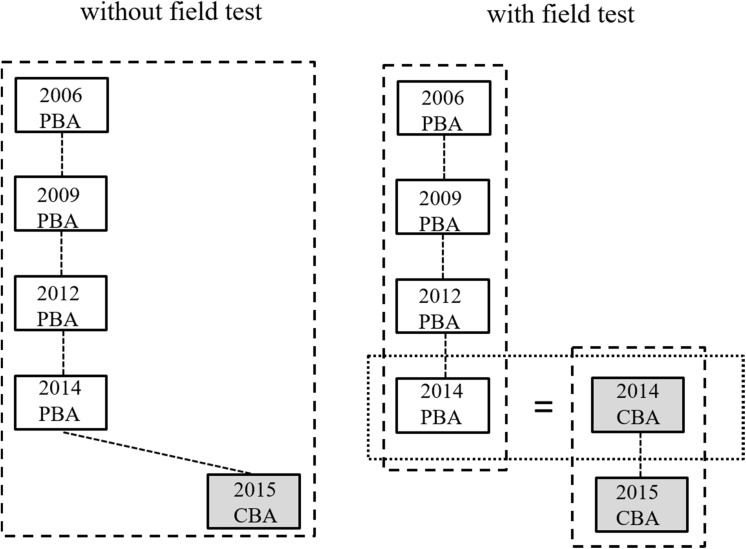
Marginal trend estimation for science without consideration **(left)** and with consideration **(right)** of the data of the German field test study of 2014.

Similar to the international approach, the study in which a competency domain was a major domain for the first time (science: PISA 2006, mathematics: PISA 2003, reading: PISA 2000) was chosen as the reference for the trend estimates. To handle a possible mode effect, two scaling strategies can be distinguished. In the first approach, the field test 2014 was not included in the scaling. This strategy is shown for the domain of science on the left side of Figure 1. For marginal trend estimation, it was assumed that there was no average mode effect for the items that were administered in CBA mode (in PISA 2015) and in (at least) one of the earlier studies. This strategy was applied to all items as well as to the subset of items declared to be invariant by the OECD.

In the second approach, the comparability of the CBA and PBA items was investigated using the field test study (see right side of Figure 1). By assuming randomly equivalent groups in the field test, differences in test performance between the two groups can only be attributed to differences between items in CBA and PBA mode. The items of the previous PISA studies (up to and including 2012) in a competency domain were linked to the items of the field test in PBA mode. Similarly, the PISA 2015 items (in CBA mode) were linked to the field test items in CBA mode. This procedure established a common metric for all PISA studies in a competency domain and adjusted for possible mode effects on all items by using the German 2014 field test as a bridge study (Mazzeo and von Davier, 2008; see also Fishbein et al., 2018, for a similar strategy in TIMSS).

Two different main scaling approaches were used: concurrent scaling and separate scaling with subsequent linking (Kolen and Brennan, 2014). In the concurrent scaling, the individual studies were treated as groups in a multigroup item response model under the assumption of invariant item parameters (Bock and Moustaki, 2006). In the separate scaling with subsequent linking, the individual studies were first scaled separately, and then the item parameters were linked either in a simultaneous linking according to the regression approach proposed by Haberman (2009) or in a stepwise linking of successive studies (chain linking; Kolen and Brennan, 2014). To estimate a competency distribution in each scaling model, 20 plausible values (Mislevy, 1991; von Davier M. et al., 2006; Adams et al., 2007) were drawn without including covariates in the background model (see also von Davier et al., 2019b, for a similar approach). All scaling models and analyses based on plausible values were conducted using student sampling weights. In total, the following 12 methods were obtained with additional consideration of the choice of a 1PL or 2PL model as the scaling model (see Table 3 for an overview).

**TABLE 3:** Overview of different linking approaches.

		IRT Model		Scaling		Linking	
	Method	1PL	2PL	conc	sep	Haber	chain
Without field test (all items)	C1	x		x			
	C2		x	x			
	H1	x			x	x	
	H2		x		x	x	
	S1	x			x		x
Without field test (invariant items)	C1I	x		x			
	C2I		x	x			
	H1I	x			x	x	
	H2I		x		x	x	
With field test	C1F	x		x			
	H1F	x			x	x	
	S1F	x			x		x

*1PL = 1PL model; 2PL = 2PL model; conc = concurrent scaling; sep = separate scaling; Haber = Linking based on the Haberman method; chain = chain-linking approach. An entry in the “linking” columns is only made for the separate scaling approaches.*

In method C1, concurrent scaling was performed according to the 1PL model, without using the German field test study, based on all items. Method C2 was based on concurrent scaling according to the 2PL model (generalized partial credit model; Muraki, 1997) without consideration of the field test. In method H1, a separate scaling according to the 1PL model was carried out. Subsequently, the item difficulties obtained from the scaling were linked with the regression approach of Haberman (2009). The link items of all PISA studies – except the German field test for PISA 2015 – were used. Method H2 is similar to method H1, except that the separate scaling was conducted using the 2PL model. In the next step, item difficulties and item slope parameters were linked according to the Haberman method (2009). In method S1, a separate scaling according to the 1PL model was also performed. However, the linking of the different studies was then carried out in a chain-linking approach (Kolen and Brennan, 2014). In each linking step of the chain, a subsequent study (e.g., PISA 2009) was linked to a previous study (e.g., PISA 2006) with mean–mean linking.

In the C1I method, in contrast to the C1 method, only the items identified as invariant by the OECD were linked in the concurrent scaling approach. The non-invariant items received item parameters in 2015 (under CBA) that were allowed to be different from the previous studies (until 2012 under PBA). The C2I method is similar to the C1I method, except that a 2PL model was used for the concurrent scaling. It thus largely corresponds to the analysis strategy used in the PISA 2015 study (see OECD, 2017). In the H1I method, as in the H1 method, linking was carried out according to the Haberman method (Haberman, 2009), whereby only the items identified as invariant by the OECD were used in the studies. This procedure largely corresponds to the analysis strategy used in the PISA studies from 2000 to 2012. The H2I method proceeded in the same way as the H1I method, except that a 2PL model was used for separate scaling.

In the C1F method, concurrent scaling was performed using the 1PL model, taking the German field test study into account. For all items used in the PBA mode (studies 2000–2012 as well as the 2014 German field test), invariant item difficulties were assumed in the scaling. Invariance was also assumed for the items used in the CBA mode (German field test 2014 and PISA 2015). A common metric for all studies was established by specifying the same competence distribution for the PBA and CBA samples in the field test. Due to the small sample sizes per item in the field test, only the 1PL model was used. In the H1F method, a separate scaling using the 1PL model was carried out, and item difficulties were linked according to Haberman’s regression approach, taking the German field test study into account. The regression approach was used separately for items in PBA mode and in CBA mode. The common metric was obtained again by assuming equivalent competence distributions for the PBA and CBA samples in the field test (see right side of Figure 1). In the S1F method, first, a separate scaling was conducted using the 1PL model. Subsequently, chain linking was performed for both the items in PBA mode and CBA mode. As in the H1F method, a common metric was established by assuming equivalent competence distributions for the PBA and CBA samples in the field test. As in Study 1, the sample sizes of the field test proved to be too small to conduct a linking based on a 2PL model.

The computation of standard errors for the trend estimates followed the analysis strategy used by the OECD. The uncertainty associated with the sampling of students was assessed using a balanced repeated replication (BRR) method based on the original data set of 80 replication zones (OECD, 2017). Link errors that assess the uncertainty that is associated with the selection of items were determined by a jackknife of items, using testlets as jackknife zones as in the field test (28 testlets in science, 38 testlets in mathematics, and 24 testlets in reading). The total standard error (*SE*_*tot*_) was calculated by adding the squared standard errors associated with student sampling and the link error and then taking the square root (OECD, 2017).

### Results

In order to achieve a better understanding of the marginal trend estimates, it is instructive to first look at the item difficulties that were obtained from a separate scaling of the German samples using a 1PL model (see Table 4). For this analysis, the abilities for each study were centered (i.e., the means of the ability distribution equaled zero), so that changes in the mean difficulties were associated with a change in the mean ability. Items administered in the same PISA studies were classified into item groups. In mathematics, for example, two item groups can be distinguished between (M1A and M1B). The 31 items of group M1A were used in all studies between PISA 2003 and 2015. It is evident that the average item difficulty of this group decreased significantly from 2003 to 2012 (−0.18 to 0.01 = −0.19 logits), indicating that there was a positive trend in mathematics over the 9 years. By contrast, the mean item difficulty increased from 2012 to 2015 in both item group M1A and item group M1B (comprising only items used in 2012 and 2015), revealing a drop in mathematical performance over this period. However, when interpreting this decrease from PISA 2012 to 2015, it is essential that the difference between the mean item difficulties of the PBA mode and the CBA mode (German field test 2014) are taken into account. It is noticeable that, after an adjustment of the differences in difficulties between the modes [e.g., for PISA 2015 and item group M1A: −0.07 + (−0.10) = −0.17], the differences in the mean item difficulties between 2012 and 2015 almost completely vanished.

**TABLE 4:** Item difficulties of link items from the 1PL Model.

Domain	Item group	#Items	2000 PBA	2003 PBA	2006 PBA	2009 PBA	2012 PBA	2015 CBA	2014 PBA vs. CBA
Science	S2A	52	−	−	−0.40	−0.41	−0.49	−0.34	−0.24
	S2B	25	−	−	−0.29	−	−	−0.13	−0.23
Mathematics	M1A	31	−	0.01	−0.11	−0.14	−0.18	−0.07	−0.10
	M1B	35	−	−	−	−	−0.08	0.17	−0.25
Reading	R1A	24	−0.34	−0.32	−0.41	−0.62	−	−0.53	−0.34
	R1B	11	−0.97	−	−	−1.09	−	−1.10	−0.37
	R2A	39	−	−	−	−0.57	−0.66	−0.56	−0.07
	R2B	8	−	−	−	0.00	−	−0.18	0.12

*Item group = Label for subset of link items that were used in different PISA studies; #Items = Number of corresponding items in an item group; 2014 PBA vs. CBA = Difference between item difficulties in PBA and CBA modes in the field test study for PISA 2015, conducted in 2014: Negative values in the column furthest the right indicate that items are easier in the PBA mode.*

Furthermore, we found that the trend estimates depended on item groups. The dependency was most pronounced for the trend estimates from 2000 to 2009 in the domain of reading. For item group R1A, for example, the difference in item difficulties between 2000 and 2009 was −0.28, whereas for item group R1B, the difference was considerably lower, at −0.12, thus indicating that the increase in item difficulty was more substantial for item group R1A than for item group R1B. This discrepancy has a direct consequence for the results obtained by different linking methods. Because only items occurring in subsequent studies were used in the stepwise linking (method S1) from 2000 to 2009 via the 2003 and 2006 studies, the trend estimation in the stepwise approach was solely determined based on item group R1A. With a joint linking approach (method H1), however, both item groups (R1A and R1B) were included in the trend estimation, so that a smaller trend estimate was shown in method H1 than in method S1.

In Table 5, the results of trend estimates for science (mean and trend for 2012–2015) are shown. The row labeled as “original” contains the means that were provided in the international OECD reports for the PISA studies. It is evident that the results for the concurrent scaling with the C1 and C2 methods based on all items mostly agree with the international findings (e.g., for PISA 2015, 509 points were reported for Germany, and the analyses with the German samples each yielded 508 points). The separate scaling with subsequent linking also led to a similar trend estimate in PISA 2015, both in the stepwise approach (method S1) and according to Haberman’s regression approach (methods H1 and H2) with 511, 506, and 501 points, respectively. It should also be emphasized that the trend estimates were similar for the 1PL model and the 2PL model. The methods that were based only on the invariant items determined by the OECD (methods C1I, C2I, H1I, and H2I) led to slightly higher trend estimates, with 506 to 513 points. However, the trend was still negative.

**TABLE 5:** Trend estimation for science in Germany.

	Method	2006	2009	2012	2015	Trend 2012 → 2015			
						*Est*	SE_*tot*_	SE_*p*_	SE_*i*_
Original		516	520	524	509	−15	5.6	4.0	3.9
Without field test (all items)	C1	516	519	523	508	−15	5.8	3.0	5.0
	C2	516	518	524	508	−16	5.8	3.0	5.0
	H1	516	515	522	506	−16	6.7	4.2	5.2
	H2	516	516	522	501	−21	7.0	4.2	5.5
	S1	516	517	524	511	−13	6.7	4.2	5.2
Without field test (invariant items)	C1I	516	519	523	513	−10	5.8	3.0	5.0
	C2I	516	519	524	513	−11	5.8	3.0	5.0
	H1I	516	517	523	513	−10	7.1	4.2	5.7
	H2I	516	518	524	506	−18	6.8	4.2	5.3
With field test	C1F	516	520	524	528	4	5.8	3.0	5.0
	H1F	516	516	522	528	6	8.3	4.2	7.2
	S1F	516	517	524	531	7	5.8	3.0	5.0

*SE_tot_ = standard error due to sampling of persons and items; SE_p_ = standard error due to sampling of persons; SE_i_ = standard error due to sampling of items (link error).*

In all nine methods in which a possible mode effect was not considered at all or only on the subset of non-invariant items, a statistically significant negative trend in science performance was obtained. If, on the other hand, the trend estimates were adjusted by the mode effect (that was identified in the German field test study), the trend was slightly positive but no longer statistically significant (PISA 2015: 528, 528, and 531 points). This observation was independent of whether a concurrent (C1F) or a separate scaling (H1F and S1F) was carried out.

In contrast to the internationally reported trend, there were almost no changes in the performance in PISA 2015 compared to the results in PISA 2012 for the mathematics domain if the German field test data were used to adjust for mode effects (see Table 6). The choice of the scaling model (1PL vs. 2PL) had almost no influence on mathematics performance. It is noticeable, however, that the trend estimate based on the invariant items determined by the OECD led to a stronger correction. Furthermore, it became apparent that the national trends substantially deviated from the internationally reported means (e.g., in PISA 2006, 504 points were reported by the OECD, and in the marginal trend estimation without field test, points ranged between 512 and 517).

**TABLE 6:** Trend estimation for mathematics in Germany.

	Method	2003	2006	2009	2012	2015	Trend 2012 → 2015			
							*Est*	SE_*tot*_	SE_*p*_	SE_*i*_
Original		503	504	513	514	506	−8	5.4	4.1	3.5
Without field test (all items)	C1	503	513	515	522	510	−12	5.8	3.0	5.0
	C2	503	515	517	524	511	−13	5.8	3.0	5.0
	H1	503	512	514	521	505	−16	6.0	4.2	4.2
	H2	503	517	521	528	515	−13	6.2	4.2	4.5
	S1	503	512	514	518	503	−15	6.1	4.2	4.4
Without field test (invariant items)	C1I	503	513	515	522	521	−1	5.8	3.0	5.0
	C2I	503	515	517	524	522	−2	5.8	3.0	5.0
	H1I	503	512	514	521	512	−9	6.4	4.2	4.8
	H2I	503	516	521	528	524	−4	7.0	4.2	5.6
With field test	C1F	503	512	515	516	518	2	5.8	3.0	5.0
	H1F	503	512	514	514	515	1	7.5	4.2	6.2
	S1F	503	512	514	518	517	−1	5.8	3.0	5.0

*SE_tot_ = standard error due to sampling of persons and items; SE_p_ = standard error due to sampling of persons; SE_i_ = standard error due to sampling of items (link error).*

The findings for reading (see Table 7) were somewhat less stable (i.e., more sensitive to the choice of analysis method) compared to the domains of science and mathematics. Although the internationally reported trend from 2012 to 2015 showed almost no change, the marginal trend estimates, without consideration of the field test and based on all items, showed an apparent decrease (methods C1, C2, H1, H2, and S1). If, on the other hand, only the invariant items were selected or the field test was used, a positive trend estimate was found for reading from 2012 to 2015. Furthermore, it needs to be pointed out that the analyses of German PISA samples for reading, even in the earlier PISA studies, considerably deviated from the international trend in some cases. These deviations could be attributed to the fact that the average country DIF for Germany differed between the link items and the non-link items. Overall, however, the results of the analyses were in line with the assumption that the choice of scaling model (1PL vs. 2PL model) had only a small influence on the trend in reading. Controlling for possible mode effects (both by restricting them to invariant items or by adjusting them by the mode effect determined in the field test) led to a slightly positive performance trend.

**TABLE 7:** Trend estimation for reading in Germany.

	Method	2000	2003	2006	2009	2012	2015	Trend 2012 → 2015			
								*Est*	SE_*tot*_	SE_*p*_	SE_*i*_
Original		484	491	495	497	508	509	1	6.7	4.1	5.3
Without field test (all items)	C1	484	479	488	504	510	504	−6	5.8	3.0	5.0
	C2	484	484	492	502	506	501	−5	5.8	3.0	5.0
	H1	484	478	487	501	509	502	−7	6.6	4.2	5.0
	H2	484	473	478	491	504	495	−9	9.9	4.2	9.0
	S1	484	482	490	510	516	505	−11	8.0	4.2	6.8
Without field test (invariant items)	C1I	484	478	488	507	513	523	10	5.8	3.0	5.0
	C2I	484	483	491	506	511	521	10	5.8	3.0	5.0
	H1I	484	477	486	504	512	517	5	7.3	4.2	5.9
	H2I	484	473	477	496	508	510	2	11.1	4.2	10.2
With field test	C1F	484	480	489	499	501	512	11	5.8	3.0	5.0
	H1F	484	479	488	499	505	516	11	9.2	4.2	8.2
	S1F	484	482	490	510	516	528	12	5.8	3.0	5.0

*SE_tot_ = standard error due to sampling of persons and items; SE_p_ = standard error due to sampling of persons; SE_i_ = standard error due to sampling of items (link error).*

**FIGURE 2:**
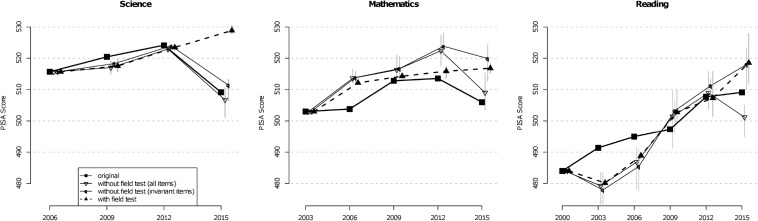
Original trend estimates and several marginal trend estimation approaches for science, mathematics, and reading. For the marginal approaches, the average of all approaches is displayed (e.g., for “with field test”: average of means of methods C1F, H1F, and S1F). The vertical gray bars are defined by the corresponding minimum and maximum.

Figure 2 shows how different strategies for considering mode effects change the marginal trend estimates for all three domains. The means and the minimum and maximum (indicated by vertical gray bars) of three method groups are depicted: methods that did not include the field test and used all items for linking, methods that did not include the field test and used only the invariant items, and methods that incorporated the field test. For science, it is apparent that only the methods that took the field test data into account led to a positive trend across all four studies and that, otherwise, there were virtually no differences between the original and marginal trends before 2015. Furthermore, there was also a positive trend in mathematics and reading when the field test was taken into account in the marginal trend estimate. However, there were stronger deviations between original and marginal trend estimates in these two domains.

## Discussion

International large-scale assessment studies in education aim to examine the performance of education systems in an international comparison. Trend estimates, which are intended to reflect whether and to what extent the performance of an education system has changed, are of particular importance. In the present article, we conducted a reanalysis of the German PISA samples to examine how trend estimates can change if the test administration mode (paper vs. computer) or the scaling model (1PL vs. 2PL model) is changed over the study period. Although the choice of the scaling model had only a minor influence on the trend estimates, the analyses using the data from the German field test study suggest that the decline reported for Germany in the performance domains from 2012 to 2015 could have been caused (at least partly) by a change in the test administration mode. In the following, we will discuss the potential limitations of our findings and also reflect on other factors that could have moderated the trend.

### Results From the German Field Test Study

In the international analysis of the PISA 2015 study, the OECD did not carry out a country-specific analysis of the field test data but instead pooled the individual country data for further analysis. Item analyses and mode effects were then examined based on this comprised sample (OECD, 2017; see also Jerrim et al., 2018a). It should be noted that country-specific samples of the field test were relatively small so that conclusions about mode-by-country interactions may not be very trustworthy. However, despite the relatively small sample in the German field test data, the mode effects turned out to be statistically significant. Jerrim et al. (2018a) investigated mode effects in the same PISA field test for Germany, Ireland, and Sweden. Although a slightly different analytical approach was used, the effect sizes of the mode effects that were reported by Jerrim et al. (2018a) match our results very closely. It should also be added that the generalizability of findings from the field test study may also be limited by the fact that its primary use in many countries was to check the test administration procedures (e.g., estimation of testing time, the feasibility of computer-based testing, etc.). Therefore, it could be argued that the field test is not directly comparable with the main PISA 2015 study in terms of the conditions of administration. It could thus be the case that mode effects were slightly overestimated and that, under more realistic conditions, mode effects would diminish. In our study, the size of mode effects in the field test was used to adjust the marginal trend estimate for Germany. Because similar mode effects for Ireland and Sweden were found in Jerrim et al. (2018a), we suppose that adjusted marginal PISA trends would also likely differ from their original trends that are included in the official PISA reports. If the mode effects were less pronounced in a future replication study of the field test, the trend estimates for Germany would be adjusted to a smaller extent.

### Differences Between Original and Marginal Trend Estimates for Germany

In our reanalyses of the German PISA studies, differences between the original and marginal trends were revealed, especially for mathematics and reading. When interpreting these differences, it should be emphasized that our analyses deviated in some technical details from the international approach. In contrast to the international analysis, we did not consider any further covariates in the background model when drawing plausible values (see also Jerrim et al., 2018b; von Davier et al., 2019b). Furthermore, our analyses were limited to subsamples of students to whom items in a respective domain were administered (von Davier et al., 2019b). In the international analysis of PISA, plausible values are also generated for students to whom no items in a domain were administered (due to the multimatrix test design in PISA).

Several scholars have argued that only original trends can be used to compare trend estimates across countries. Their main argument is that a large subset of items must possess invariant item parameters in order to ensure the comparability of countries. Alternatively, it could be argued that marginal trend estimation also establishes a common metric across countries by placing the same identification constraints upon the item parameters for each country. In line with this reasoning, it has been shown that both original and marginal trend estimation provide unbiased estimates (see Sachse et al., 2016) but that marginal trends are more robust to the choice of link items (Carstensen, 2013) and that they are more efficient than original trends (Sachse et al., 2016; Sachse and Haag, 2017; Robitzsch and Lüdtke, 2019). Hence, we believe that marginal trend estimates also allow cross-national comparisons and that the estimation of marginal trends should receive more emphasis in large-scale assessment studies. For example, marginal trends could be reported as an additional validation of original trend estimates (see also Urbach, 2013). Nonetheless, the marginal trend analyses in this article are not intended to fully replace the internationally reported original trend.

Since PISA 2015, item-by-country interactions have been allowed in the scaling model by allowing for the partial invariance of item parameters (OECD, 2017; see Oliveri and von Davier, 2011; von Davier et al., 2019b). In previous PISA studies, items were only removed from scaling for a particular country in the case of technical issues (e.g., translation errors; OECD, 2014). Therefore, comparisons of country pairs could depend on different item sets, and it could be argued that one is comparing apples with oranges (Kuha and Moustaki, 2015; Goldstein, 2017; Jerrim et al., 2018b). However, other scholars claim that the partial invariance approach provides a fairer comparison of countries (Oliveri and von Davier, 2014). As an alternative, model misfit can be modeled by an additional stochastic component in the item response model which increases standard errors of estimated parameters (Wu and Browne, 2015).

Furthermore, our analyses showed how sensitive trend estimates are to changes in the test administration mode. Possible mode effects in the original trend could have led to substantial declines in performance both in the international trend and in other participating countries in PISA 2015. Our findings illustrate the need for further research in national studies focused on mode effects (Jerrim et al., 2018a; Feskens et al., 2019).

### Choice of Item Response Model in Large-Scale Assessments

In the present article, we investigated the sensitivity of trend estimates to the choice of either the 1PL or the 2PL model. One argument frequently made by proponents of the 2PL model is that a 2PL model shows a better fit to PISA data than the 1PL model (OECD, 2017). However, using the rationale of the best-fitting model, a 3PL model for multiple-choice items, a 4PL model that also contains an upper asymptote smaller than one (Culpepper, 2017), or even a non-parametric item response model (Xu et al., 2010) could be superior to the 2PL model in terms of model fit. Hence, the choice of an item response model does not seem to be mainly driven by model fit, but more by the preferences of research groups or by historical conventions. However, the use of a 1PL model could be defended because each item gets the same weight in the scaling model. This approach stands in contrast to a more data-driven weighting of items in the 2PL or 3PL model, where more “reliable” items possess larger weights in the scaling model. It can be argued that the equal weighting of items could lead to a better alignment of the test framework than a weighting of items determined by the fit of the psychometric model (Brennan, 1998). In that case, an appropriate scaling model cannot be chosen merely by a statistical model comparison, that is, it is not a purely empirical question. Therefore, the model choice must be defended on a theoretical basis (e.g., by content experts) and must determine how the individual items are to be weighted in the scaling model. In our study, we did not find substantial differences between the 1PL and the 2PL model for trend estimates. In future research, it could be investigated whether relationships of abilities with covariates depend on the choice of the scaling model.

We would like to point out that the psychometric model chosen in a large-scale assessment study is almost always misspecified, and distribution parameters (means and standard deviations) of the competence distributions of countries and item parameters are defined as best approximations with respect to the Kullback–Leibler information (White, 1982; Kuha and Moustaki, 2015; but see also Stefanski and Boos, 2002; Buja et al., 2019). Overall, we argue that the criterion of model fit should not play the primary role in choosing a psychometric model (Brennan, 1998) because validity considerations are more important in large-scale assessment studies (Reckase, 2017; Zwitser et al., 2017). The use of misspecified item response models typically impacts the outcomes of linking several studies and trend estimates (Martineau, 2006; Zhao and Hambleton, 2017).

### How Can Paper-Based and Computer-Based Tests Be Linked?

In PISA 2015, mode effects were taken into account when estimating trends by allowing some unique item parameters to exist for items administered on a computer (OECD, 2017; von Davier et al., 2019a). In a 2PL model, item loadings were assumed to be invariant between the CBA and PBA modes, while item intercepts were allowed to differ between modes for some items. Hence, it was possible to identify a subset of items from the field test that had invariant item parameters across modes (OECD, 2017; see also Kroehne et al., 2019a). It is important to emphasize that these items were absolutely invariant in the sense that they had the same item loadings and item intercepts, but average ability differences were not controlled for. Our findings, as well as other results in the literature (Jerrim et al., 2018a; Kroehne et al., 2019a), indicate that a small mode effect favoring the PBA mode remains for these invariant items and that this mode effect has the potential to distort reliable trends at the level of all participating countries in PISA. It could be the case that the identification of invariant items based on non-significance for the difference of item parameters caused the remaining mode effect. However, it would be feasible to include an additional optimization constraint into the search for invariant items to ensure that mode effects cancel out on average for these invariant items.

In TIMSS, there was also a recent switch from the PBA to CBA administration mode. In that study, a bridge study was used, and PBA and CBA items were linked by assuming equivalent groups (i.e., equal distributions due to random allocation of administration mode; Fishbein et al., 2018). In that approach, no invariant item parameters are assumed, and mode effects are allowed to be item-specific for all items. Compared to PISA, TIMSS offers the opportunity of studying mode-by-country interactions by employing extension samples in the main study (Fishbein et al., 2018).

If the assumptions of the corresponding item response models are fulfilled, both the PISA and TIMSS linking approaches can guarantee unbiased trend estimates even when the administration mode is changed. The PISA approach is more parsimonious than the TIMSS approach because fewer item parameters are required due to invariance assumptions. On the other hand, the TIMSS approach could be seen as being more robust because it does not rely on such strong model assumptions. We believe that invariant items do not necessarily have to exist in order to ensure the comparability of CBA and PBA modes. Moreover, it could be argued that the trend established by the PISA approach poses a threat to validity because the linking is only achieved based on items that did not differ between CBA and PBA. As a consequence, the PISA approach provides trend estimates that are prone to construct underrepresentation because it has to be shown that the non-invariant items, which are removed from trend estimates, are irrelevant to ensure construct representation (i.e., these items are construct-irrelevant; see Camilli, 1993).

### Should Mode Effects Be Adjusted at All?

The adjustment of trend estimates for possible mode effects is motivated by the goal of providing stable trend information for countries participating in large-scale assessment studies. The adjusted trend extrapolates a trend for a country under the scenario that a PBA test version had been continually administered. However, it could be argued that a test score achieved in the context of a large-scale assessment study is always partly determined by the mode of administration. As the use and importance of computers increases in society and education, it seems more relevant to assess competencies in CBA than in PBA mode, and it could therefore be argued that it is not necessarily of interest how a trend would continue if it were based on a PBA. Accordingly, future research should investigate potential sources for mode effects, such as mode-related speed differences (Kroehne et al., 2019b).

**FIGURE 3:**
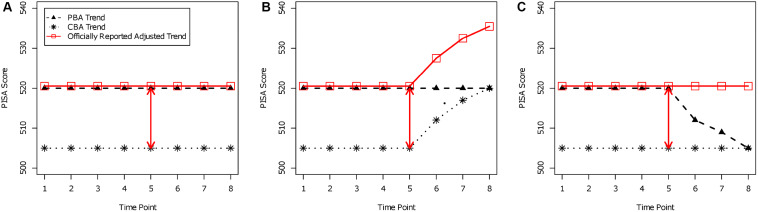
Trends for paper-based assessment (PBA), computer-based assessment (CBA), and officially reported adjusted trends. **(A)** Time-constant mode effect; **(B)** disappearing mode effect by performance increase in CBA; **(C)** disappearing mode effect by performance decrease in PBA.

In addition, trend estimation in PISA and TIMSS uses only one snapshot in time to assess the mode effect. However, if the mode effect varies across time, conclusions about the competence trend for countries can be distorted. In Figure 3, possible trends are depicted as a thought experiment. Assume that a large-scale assessment study is administered at eight time points. Until the fourth time point, test scores are presented in the PBA mode (i.e., PISA 2000 to PISA 2012). At the fifth time point, the mode effect of the switch from PBA to CBA is assessed (in PISA 2015). The test is administered in CBA mode starting with the fifth time point, but the officially reported trend estimates take the mode effect of the fifth time point into account (since PISA 2015). In the three panels of Figure 3, different constellations of a time-varying mode effect are displayed. The PBA trend, the CBA trend, and the officially reported trend are depicted in each panel. In all three constellations, the mode effect was 15 PISA points at the fifth time point (PBA: 520 points; CBA: 505 points). In panel A, the mode effect favors PBA with 15 PISA points. In this case, it seems plausible that the adjustment of the mode effect is legitimated because both the PBA and the CBA trend appear to be constant, and the officially reported adjusted trend correctly takes this into account. In panel B, it can be seen that the PBA trend is constant. However, there is an increase in the CBA mode, and at the eighth time point, the mean for the CBA mode equals the PBA mean, implying that the mode effect disappears. Still, the adjusted trend shows an increase in performance because a constant adjustment of 15 points (i.e., mode effect) is added. Obviously, the adjusted trend would provide a distorted picture of the actual performance of a country. In panel C, there is a decreasing trend in the PBA mode, but the trend in the CBA mode remains constant. At the eighth time point, the mode effect disappears as in panel B, and the adjusted trend shows a constant trend. However, one could also argue that the actual performance of a country also drops in the PBA mode to the performance in the CBA mode and that the officially reported trend estimates provide a distorted picture of the trend in competencies. These data constellations illustrate that there are reasonable arguments for not adjusting trend estimation for test administration mode if long-term trends are to be reliably estimated. As an alternative, one could repeatedly assess the mode effect at a later time (e.g., at the eighth time point) in order to check whether the mode effect changes over time. Furthermore, the adjustment of the trend needs to be time-specific.

Of course, adjusted trends have their merit in short-term trends (e.g., from PISA 2012 to PISA 2015 or 2018). We want to emphasize that these cautions also apply for the recent PISA approach that uses invariant items for linking. Items were identified as invariant just with respect to one time point (i.e., the fifth time point in Figure 3), and it cannot be ensured that the mode effects of these items are invariant across all possible time points of the study.

## Conclusion

PISA 2015 switched from PBA to CBA. In addition to this change, the scaling model was also changed. It is, therefore, vital to investigate whether both changes affected national trend estimates. We used the German data from PISA 2000 to PISA 2015 to investigate both questions in more detail. The main findings for Germany are as follows. First, the change of the scaling model was not related to the decline in mathematics and science. Second, based on the field test data from 2014, we found that PISA items are, on average, more difficult if they are administered on a computer instead of on paper (i.e., a mode effect). Third, the negative impact of computer administration on the performance of 15-year-olds was visible in all three domains (science, mathematics, and reading). Fourth, assuming that the mode effects we found in PISA 2015 were of the same size as those found for German students as in the field test study 2014, had these mode effects been controlled for, the trend estimates between 2012 and 2015 for mathematics and science performance would have remained unchanged and would have improved slightly in reading. The internationally reported trend estimates between PISA 2012 and 2015 should, therefore, be interpreted with some caution.

## Author’s Note

This article builds on and extends an earlier article published in German by Robitzsch et al. (2017).

## Data Availability Statement

The datasets generated for this study will not be made publicly available the international PISA datasets are available from the OECD website. The PISA field test data (conducted in 2014) cannot be made publicly available. Requests to access the datasets should be directed to the corresponding author.

## Ethics Statement

The studies involving human participants were reviewed and approved by OECD. Written informed consent to participate in this study was provided by the participants’ legal guardian/next of kin.

## Author Contributions

All authors listed have made a substantial, direct and intellectual contribution to the work, and approved it for publication.

## Conflict of Interest

The authors declare that the research was conducted in the absence of any commercial or financial relationships that could be construed as a potential conflict of interest.
